# Effect of latent membrane protein 1 expression on overall survival in Epstein-Barr virus-associated cancers: a literature-based meta-analysis

**DOI:** 10.18632/oncotarget.4906

**Published:** 2015-08-18

**Authors:** Yu-Pei Chen, Wen-Na Zhang, Lei Chen, Ling-Long Tang, Yan-Ping Mao, Wen-Fei Li, Xu Liu, Guan-Qun Zhou, Ying Sun, Tie-Bang Kang, Mu-Sheng Zeng, Na Liu, Jun Ma

**Affiliations:** ^1^ Sun Yat-sen University Cancer Center; State Key Laboratory of Oncology in South China; Collaborative Innovation Center for Cancer Medicine, Guangzhou, People's Republic of China

**Keywords:** LMP1, EBV, cancer, survival, meta-analysis

## Abstract

Latent membrane protein 1 (LMP1) is identified as the main transforming oncoprotein of Epstein-Barr virus (EBV). LMP1 is frequently expressed in a variety of EBV-associated cancers, including nasopharyngeal carcinoma (NPC), non-Hodgkin lymphoma (NHL), Hodgkin disease (HD), and gastric cancer (GC). However, due to conflicting results, the prognostic value of LMP1 expression on clinical outcomes in EBV-associated cancers remains unclear. We performed a meta-analysis on 32 studies with a total of 3752 patients to explore the association between LMP1 expression and overall survival (OS) in EBV-associated cancers. Overall, LMP1 expression was significantly associated with poorer OS (hazard ratio, HR = 1.51, 95% confidence interval, CI, 1.13–2.03), irrespective of cancer type. Further analyses showed that LMP1 expression correlated with poorer OS in NPC (HR = 2.48, 95% CI, 1.77–3.47) and NHL patients (HR = 1.83, 95% CI, 1.07–3.15), but not in HD patients (HR = 0.98, 95% CI, 0.60–1.62) or GC patients (HR = 0.70, 95% CI, 0.44–1.12). Subgroup analyses indicated that the age and geographical factors seemed to have an effect on the clinical outcomes of HD patients with positive LMP1 expression. In conclusion, LMP1 expression can be used as a prognostic biomarker in NPC, NHL, and certain HD patients. This data suggests that novel therapies targeting LMP1 may improve clinical outcomes for EBV-associated cancer patients.

## INTRODUCTION

Epstein-Barr virus (EBV) is a ubiquitous tumorigenic human herpes virus carried in more than 90% of adult populations worldwide [1]. EBV has been implicated in a number of human malignancies of either epithelial or lymphoid origin, including nasopharyngeal carcinoma (NPC), lymphoma, and gastric cancer (GC) [2]. Different patterns of latent EBV gene expression are observed in these tumors, which can alter the phenotype and cause oncogenic transformation of EBV-infected cells. Of these gene products, latent membrane protein 1 (LMP1) from EBV has been identified as the main transforming oncoprotein of EBV [3].

Expression of LMP1 in EBV-associated cancers is associated with the regulation of proliferation, immortalization, invasion, and angiogenesis of tumor cells [1–4]. LMP1 is an integral membrane protein comprising three domains: a short cytoplasmic N-terminus, six transmembrane spanning regions, and a large cytoplasmic C-terminal tail [4]. It activates the tumor necrosis factor receptor (TNFR) signaling pathway through recruitment of TNFR-associated factors and other adaptor proteins, and stimulates or inhibits several other signaling pathways, including NF-kB and PI3-K/Akt [3, 4]. Through these signaling events, LMP1 has been shown to transform rodent fibroblast cells, cause B lymphocyte immortalization *in vitro*, and induce hyperplasia in transgenic mice [5]. In addition, as LMP1 resembles CD40 in its functionality, it can be partially used to substitute for CD40 *in vivo*, to enhance B lymphocyte proliferation [6]. Therefore, LMP1 is attracting considerable attention as a potential prognostic biomarker and novel therapeutic target.

Despite the clinical implication of LMP1 expression, its prognostic value on clinical outcomes across different EBV-associated cancers remains unclear. While some studies indicate that LMP1 expression is positively associated with cervical lymph node metastasis [7] and is an unfavorable prognostic factor in NPC [8, 9], others found no significant association [10, 11]. In addition, LMP1 expression is suggested to be an unfavorable prognostic factor for non-Hodgkin lymphoma (NHL) patients but has no effect on the overall survival (OS) of Hodgkin disease (HD) patients [12–16]. Other studies indicate that certain epidemiologic factors (e.g., age, geographical factors, socioeconomic status, and so on) might influence the prognostic impact of LMP1 expression in lymphomas [12–16].

Due to these conflicting results, a comprehensive analysis of the prognostic effects of LMP1 is warranted. We conducted a meta-analysis of the literature to explore the association between LMP1 expression and OS among patients with different types of EBV-associated cancers. An improved understanding of this issue will enhance rational development of more targeted EBV-associated cancer therapy, which has important public health and clinical implications.

## RESULTS

### Eligible studies

**Figure 1:**
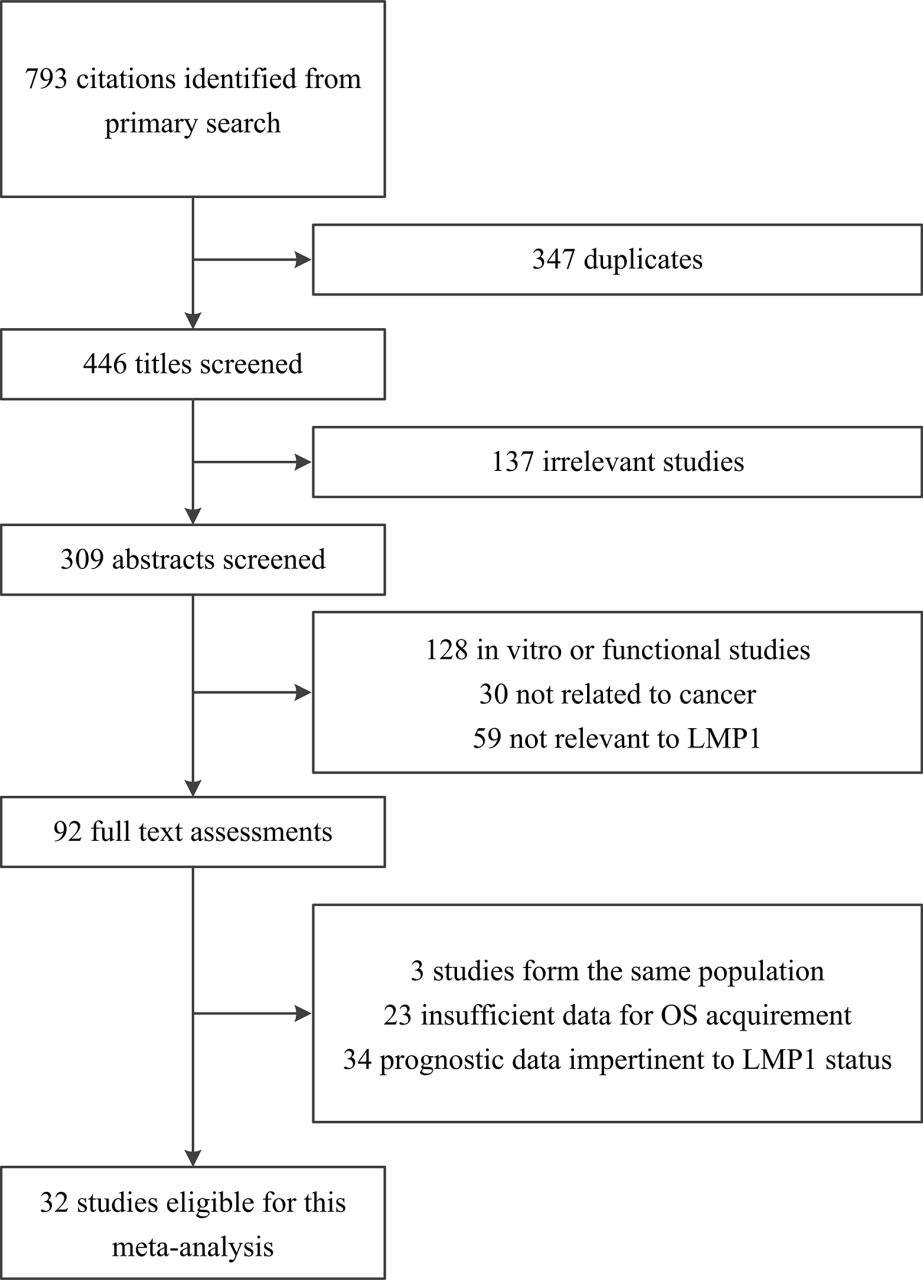
Flow chart showing the selection of the 32 studies included in the meta-analysis LMP1, latent membrane protein 1; OS, overall survival.

A total of 793 citations were identified after our initial search. After the selection procedure, 32 studies that met our inclusion criteria were included in this meta-analysis [8–11, 13–40]. Figure 1 summarizes the flow chart of study selection. Of these 32 studies, eight (25%) evaluated NPC [8–11, 17–20], 23 (72%) evaluated lymphoma [13–16, 21–39], and one (3%) evaluated GC [40]. Of the 23 studies evaluating lymphoma, nine (39%) focused on NHL [13, 21–28], while the remaining 14 (61%) focused on HD [14–16, 29–39]. Three studies were prospectively conducted [14, 36, 39], and the remaining studies used retrospective cohort designs. The characteristics of all included studies are presented in Table 1.

**Table 1:** Characteristics of the 32 studies included in the meta-analysis

First author, year	Country	Period	Histology	Detection method	Cutoff value for detection	No. of subjects (LMP1+/LMP1−)	Median/mean age (range)	Median/mean follow-up time (months)	Quality score	HR (95% CI) for overall survival
**NPC**										
Chen, 2010	China	1992–2002	NPC	IHC	IRS, ≥4	224 (141/83)	46 (14–86)	NA	8	2.06 (1.16–3.64)
Hariwiyanto, 2010	Indonesia	NA	NPC	IHC	H-score, >7	56 (27/29)	(11–70)*	NA	7	5.56 (1.87–16.50)
Kitagawa, 2013	Japan	1998–2009	NPC	IHC	Percentage, ≥10%	74 (35/39)	Median >50	45.24	7	1.26 (0.69–2.28)
Li, 2009	China	1999–2003	NPC	IHC	Percentage × staining intensity, ≥1	57 (24/33)	56.2 (22–72)	36	8	2.73 (0.91–8.17)
Sarac, 2001	Turkey	1979–1993	Un differentiated NPC	IHC	Positive: detectable	35 (10/25)	35 (5–71)	66	7	2.82 (0.88–8.98)
Song, 2007	China	2001–2003	NPC	IHC	Percentage, ≥10%	50 (25/25)	50.24*	NA	8	4.72 (1.45–15.33)
Wang, 2008	China	1999–2003	Nonkeratin NPC	IHC	Percentage, ≥25%	60 (24/36)	53 (18–79)	36–74	8	3.17 (1.37–7.31)
Zhu, 2004	China	1990–1991	Un differentiated NPC	IHC	Percentage × staining intensity, ≥1	60 (39/21)	38 (13–65)	56	8	2.80 (1.30–6.04)
**NHL**										
Cao, 2008	China	1994–2000	ENKL	IHC	Percentage, ≥ 10%	58 (47/11)	45.4 (10–78)	84	7	2.59 (1.01–6.67)
Hirose, 2006	Japan	1980–2004	PTCL	IHC	NA	43 (14/29)	63 (17–86)	14	6	1.68 (0.80–3.54)
Ishii, 2007	Japan	1990–2003	ENKL	PCR	>40 copies/ml	20 (13/7)	52.5 (28–71)	34	7	7.02 (1.91–25.73)
Kanemitsu, 2012	Japan	1996–2010	ENKL	IHC	NA	30 (22/8)	62 (27–85)	26.7	6	0.24 (0.07–0.80)
Kuze, 1996	Japan	1983–1995	BCL	IHC	NA	17 (6/11)	60 (35–82)	12	5	0.85 (0.19–3.82)
Paydas, 2008	Turkey	NA	NHL	IHC	NA	138 (20/118)	51.6 (16–82)	NA	5	3.49 (1.68–7.25)
Xu, 2009	China	1995–2005	ENKL	IHC	Percentage × staining intensity, ≥1	62 (30/32)	41 (13–79)	NA	7	1.73 (0.86–3.46)
Yamamoto,1999	Japan	1974–1994	TCL	ISH	mRNA positive in tumor cells	25 (15/10)	NA	>36	7	3.80 (0.92–15.80)
Zhao, 2005	China	2000–2004	ENKL	IHC	Positive: detectable	36 (6/30)	40 (16–71)	17.7	7	1.28 (0.40–4.02)
**HD**										
Clarke, 2001	USA	1988–1994	HL	IHC	NA	78 (51/27)^†^^‡^	(45–79)*	73	6	3.00 (1.50–6.40)
Claviez, 2005	Germany & Austria	1990–2001	HL	IHC	NA	842 (263/579)	13.7 (2.2–20.2)	58.5	7	3.00 (1.22–7.39)
Dinand, 2009	India	1991–2004	cHL	IHC	Percentage, ≥25%	122 (113/9)	8 (2–14)	48	7	0.60 (0.10–4.90)
Enblad, 1999	Sweden	1985–1988	HL	IHC	NA	117 (32/85)	45 (11–87)	130	7	2.06 (0.71–6.00)
Engel, 2000	South Africa	NA	HL	IHC	NA	36 (24/12)^‡^	8 (3–14)	4–150	7	0.08 (0.02–0.45)
Glavina-Durdov, 2001	Croatia	1980–1990	HL	IHC	NA	100 (26/74)	40 (13–84)	NA	7	0.98 (0.42–2.32)
Herling, 2003	USA& Italy & Greece	1984–2000	cHL	IHC	Positive: detectable	303 (61/242)	30*	65	7	1.11 (0.50–2.45)
Keresztes, 2005	Hungary	NA	HL	IHC	NA	109 (47/62)	31 (3–74)	83	6	2.13 (0.74–6.15)
Krugmann, 2003	Austria	1974–1999	cHL	IHC	NA	119 (31/88)	37.6 (14–83)	122	7	0.96 (0.39–2.33)
Morente, 1997	Spain	NA	HL	IHC	Positive: detectable	140 (72/68)	37.2 (5–83)	65	8	0.39 (0.17–0.92)
Murray, 1999	UK	1992–1996	HL	IHC	NA	161 (41/120)	33 (22–49)	86	6	0.71 (0.32–1.57)
Naresh, 2000	India	1984–1988	cHL	IHC	Percentage, ≥10%	110 (86/24)^‡^	22 (4–61)	57	6	0.26 (0.08–0.88)
Quijano, 2004	Colombia	1994–1998	HL	IHC	NA	57 (32/25)	(3–83)*	23.8	6	0.36 (0.08 -1.60)
Stark, 2002	UK	1991–1998	HL	IHC	NA	70 (24/46)	(60–91)*	62.5	8	3.12 (1.36–7.11)
**GC**										
Lee, 2004	Korea	1995–1996	GC	IHC	Percentage, ≥10%	343 (63/280)^‡^	55*	54	6	0.70 (0.44–1.13)

Abbreviations: cHL, classical Hodgkin lymphoma; CI, confidence interval; ENKL, extranodal NK/T-cell lymphoma, nasal type; GC, gastric cancer; HD, Hodgkin disease; HR, hazard ratio; H-score, histochemistry score; IHC, immunohistochemistry; IRS, immunoreactive score; ISH, *in situ* hybridization; LMP1, latent membrane protein 1; NA, not available; NHL, non-Hodgkin lymphoma; NPC, nasopharyngeal carcinoma; PCR, polymerase chain reaction; PTCL, peripheral T-cell lymphomas; TCL, T-cell lymphoma.

* Median/mean age or range is not available.

† The survival data was only available for patients aged older than 45 years.

‡ The positive/negative cases in these studies were positive/negative for Epstein-Barr virus encoded nuclear RNA-1 (EBER-1) as detected by *in situ* hybridization. The positive rates of LMP1 detected by IHC were 69%, 90%, 65%, and 93% in the EBER-1 positive cases in the studies by Clarke, Engel, Naresh, and Lee, respectively.

A total of 3752 patients were analyzed for LMP1 status and its relationship to disease prognosis, of which 1464 (39%) were classified as LMP1 positive. In the 32 studies, 30 (94%) investigations detected the LMP1 expression by immunohistochemistry (IHC), one (3%) used the polymerase chain reaction (PCR), and one (3%) used *in situ* hybridization (ISH). It should be noted that the primary detection method in four studies [29, 30, 37, 40] was ISH detecting EBV encoded nuclear RNA-1 (EBER-1) and the positive/negative cases in these studies were positive/negative for EBER-1 expression. In these EBER-1 positive cases, the positive rates for LMP1 expression were 69% [29], 90% [30], 65% [37], and 93% [40]. Geographically, 10 (31%) studies were conducted in Europe and North America, 20 (62%) in Asia, one (3%) in South America, and one (3%) in South Africa. The quality of the included studies, as assessed by the Newcastle-Ottawa Scale (NOS), ranged from five to eight stars, with 22 (69%) studies of high quality, and 10 (31%) of low quality (Table 2).

**Table 2:** Quality assessment of eligible studies using the Newcastle-Ottawa Scale (NOS)

First author, year	Represen tativeness of exposed cohort	Selection of non exposed cohort	Ascer tainment of exposure	Demonstration that outcome was not present at start of study	Comparability based on the design or analysis	Assessment of outcome	Follow-up long enough for outcomes to occur	Adequacy of follow-up of cohorts	Total NOS score (stars)
**NPC**									
Chen, 2010	1	1	1	1	2	1	1	0	8
Hariwiyanto, 2010	1	1	1	1	2	1	0	0	7
Kitagawa, 2013	1	1	1	1	1	1	1	0	7
Li, 2009	1	1	1	1	2	1	1	0	8
Sarac, 2001	1	1	1	1	0	1	1	1	7
Song, 2007	1	1	1	1	1	1	1	1	8
Wang, 2008	1	1	1	1	1	1	1	1	8
Zhu, 2004	1	1	1	1	1	1	1	1	8
**NHL**									
Cao, 2008	1	1	1	1	0	1	1	1	7
Hirose, 2006	1	1	1	1	1	1	0	0	6
Ishii, 2007	1	1	1	1	1	1	0	1	7
Kanemitsu, 2012	1	1	1	1	1	1	0	0	6
Kuze, 1996	1	1	1	1	0	1	0	0	5
Paydas, 2008	1	1	1	1	0	1	0	0	5
Xu, 2009	1	1	1	1	0	1	1	1	7
Yamamoto,1999	1	1	1	1	0	1	1	1	7
Zhao, 2005	1	1	1	1	0	1	1	1	7
**HL**									
Clarke, 2001	1	1	1	1	0	1	1	0	6
Claviez, 2005	1	1	1	1	0	1	1	1	7
Dinand, 2009	1	1	1	1	0	1	1	1	7
Enblad, 1999	1	1	1	1	0	1	1	1	7
Engel, 2000	1	1	1	1	1	1	0	1	7
Glavina-Durdov, 2001	1	1	1	1	1	1	0	1	7
Herling, 2003	1	1	1	1	1	1	1	0	7
Keresztes, 2005	1	1	1	1	0	1	1	0	6
Krugmann, 2003	1	1	1	1	0	1	1	1	7
Morente, 1997	1	1	1	1	1	1	1	1	8
Murray, 1999	1	1	1	1	1	1	0	0	6
Naresh, 2000	1	1	1	1	0	1	1	0	6
Quijano, 2004	1	1	1	1	1	1	0	0	6
Stark, 2002	1	1	1	1	1	1	1	1	8
**GC**									
Lee, 2004	1	1	1	1	0	1	1	0	6

Abbreviations: HL, Hodgkin lymphoma; NHL, non-Hodgkin lymphoma; NPC, nasopharyngeal carcinoma; GC, gastric cancer.

### Meta-analysis of the effect of LMP1 expression and overall survival in EBV-associated cancers

**Figure 2:**
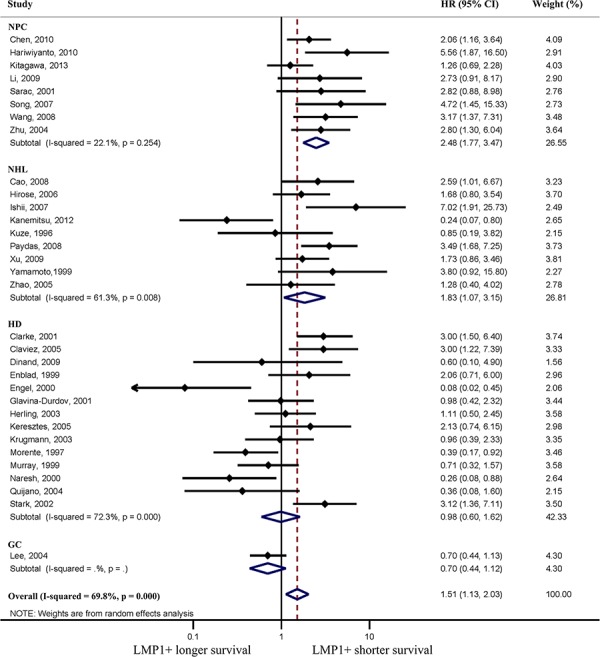
Forest plot showing association of latent membrane protein 1 (LMP1) expression and overall survival (OS) Pooled estimates of hazard ratio (HR) are based on random effects meta-analysis. Horizontal line represents 95% confidence interval (CI). HD, Hodgkin disease; NHL, non-Hodgkin lymphoma; NPC, nasopharyngeal carcinoma; GC, gastric cancer.

The positive expression of LMP1 was statistically associated with a poorer OS (hazard ratio, HR = 1.51; 95% confidence interval, CI, 1.13–2.03; Figure 2) when including all 32 studies; however, significant heterogeneity was detected (I^2^ = 70%; *P* < 0.001). In NPC patients, the pooled random-effects model showed a significantly poorer OS with positive expression of LMP1 (HR = 2.48; 95% CI, 1.77–3.47; Figure 2); no significant heterogeneity was observed (I^2^ = 22%; *P* = 0.254). The estimated HR was 2.36 (95% CI, 1.77–3.15) using a fixed effects model. In NHL patients, a significant association between LMP1 expression and poorer OS was also observed (HR = 1.83; 95% CI, 1.07–3.15), while no significant association was found in HD patients (HR = 0.98; 95% CI, 0.60–1.62). However, significant heterogeneity was found in studies on NHL (I^2^ = 61%; *P* = 0.008) and HD patients (I^2^ = 72%; *P* < 0.001; Figure 2). Only one study was included for GC, which showed that the expression of LMP1 had no significant correlation with OS (HR = 0.70; 95% CI, 0.44–1.12; Figure 2).

### Heterogeneity, sensitivity analyses, and publication bias

Significant heterogeneity was exhibited in studies on NHL and HD patients. Of the nine studies evaluating NHL, the study by Kanemitsu et al. [13] was a notable outlier in that it indicated a significantly more favorable prognosis in LMP1 positive patients (Figure 2). After excluding this study, no significant heterogeneity existed (I^2^ = 19%; *P* = 0.282), and the combined HR based on the fixed effects model of the remaining eight studies was 2.25 (95% CI, 1.61–3.13).

**Figure 3:**
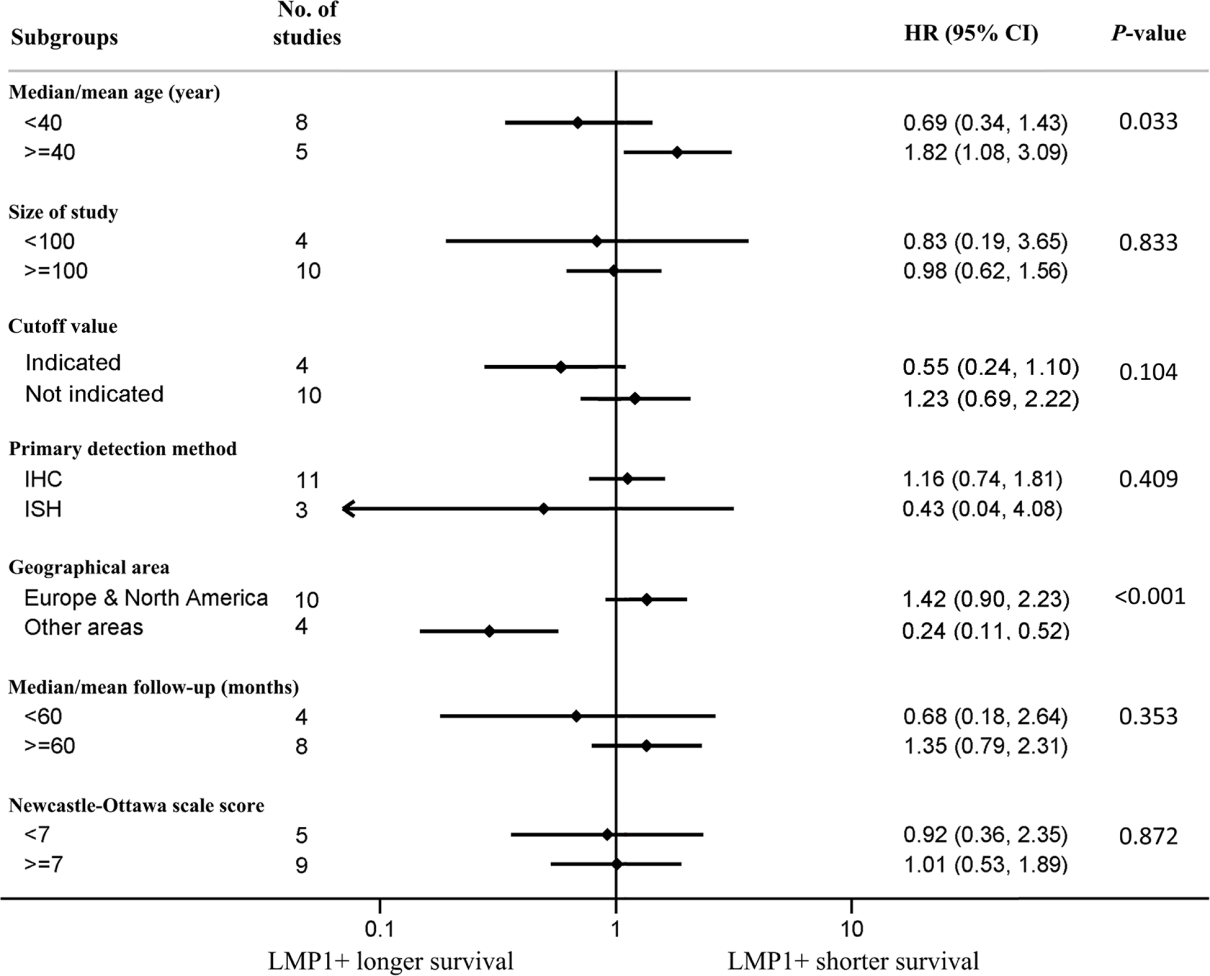
Subgroup analyses showing association of latent membrane protein 1 (LMP1) expression and overall survival (OS) according to various factors in Hodgkin disease (HD) patients Median/mean age data was not available for the study by Quijano et al. and median/mean follow-up (months) was not available for the studies by Engel et al. and Glavina-Durdov et al. Pooled estimates of hazard ratio (HR) are based on random effects meta-analysis. Horizontal line represents 95% confidence interval (CI). IHC, immunohistochemistry; ISH, *in situ* hybridization.

Figure 3 shows the results of the subgroup analyses for HD patients. The prognostic effects were similar between the five predefined subgroups according to size of study, cutoff value, primary detection methods, median/mean follow-up time, and NOS scores. However, the prognostic effects based on median/mean age and geographical area appeared discordant. The combined HR was 0.69 (95% CI, 0.34–1.43) for studies with a median/mean age <40 years, while a significantly poorer OS was associated with LMP1 expression in studies with a median/mean age ≥40 years (HR = 1.82; 95% CI, 1.08–3.09). No significant association between LMP1 expression and survival was found in patients from Europe and North America (HR = 1.42; 95% CI, 0.90–2.23), while a significantly better OS was associated with LMP1 expression in patients from other areas, such as Asia, South Africa and South America (HR = 0.24; 95% CI, 0.11–0.52). This may partly explain the substantial heterogeneity observed when examining LMP1 expression as a prognostic factor in HD patients.

**Figure 4:**
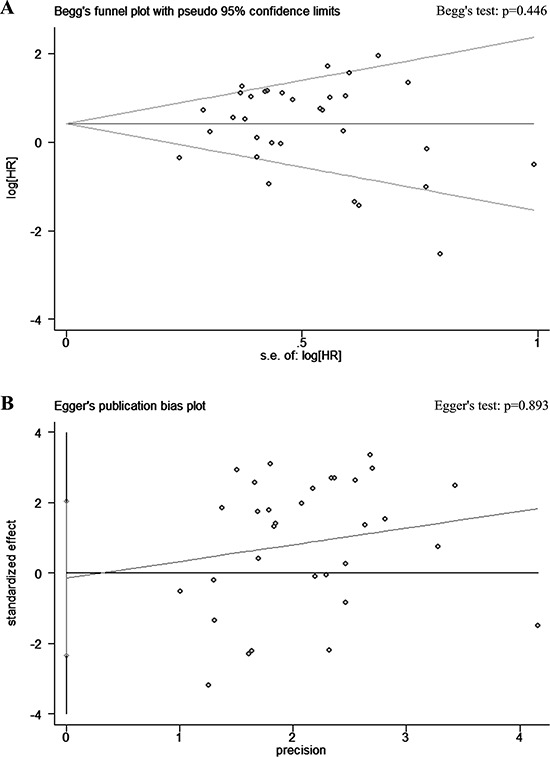
Funnel plots with A. Begg test and B. Egger test showing association of latent membrane protein 1 (LMP1) expression and overall survival (OS) in all 32 included studies Visual inspection of the Begg and Egger funnel plots did not identify substantial asymmetry. HR, hazard ratio; S.E., standard error.

Funnel plots with the Begg test and Egger test are shown in Figure 4. With all 32 included studies, visual inspection of the Begg and Egger funnel plots did not identify substantial asymmetry (*P* = 0.446 using the Begg test, and *P* = 0.893 using the Egger test), indicating that there was no evidence of publication bias detected in this study (Figure 4).

## DISCUSSION

LMP1 has been implicated in the etiology of various EBV-associated cancers. However, the direction and magnitude of the prognostic effect of LMP1 expression, and whether the outcome is consistent among different patient subgroups, remains unresolved. Here we conducted a meta-analysis of 3752 patients included in 32 studies, and found that EBV-associated cancer patients with positive expression of LMP1 had significantly poorer survival than those with negative expression. Subgroup analyses showed that LMP1 expression was a significant unfavorable biomarker in NPC and NHL, but it had no significant effect on survival of HD and GC patients. Instead, age and geographical factors seemed to affect the clinical outcomes of HD patients with positive LMP1 expression.

NPC is particularly common in south China, reaching a peak incidence of 20–50 per 100 000 males, and shows the most consistent worldwide association with EBV [3, 41]. Our meta-analysis found that LMP1 expression was a strong risk factor for the prognosis of NPC; the risk of death was 2.48 fold higher in NPC patients with positive expression of LMP1. This was the highest risk of death among all EBV-associated cancers studied (i.e., NPC, NHL, HD, and GC).

There are a few possible underlying mechanisms involved in the association between LMP1 expression and poorer OS in NPC patients. LMP1 contributes to invasion and metastasis by modulating cell-matrix interactions through induction of matrix metalloproteinases (MMPs), and downregulation of various metastasis suppressors [4, 8]. In addition, LMP1 modulates key tumor suppressor genes and micro-RNAs, thereby imparting resistance to apoptosis [4, 8]. Finally, LMP1 may trigger overall tumor cell growth, motility and invasiveness through the activation of NF-kB, JNK/p38-SAPK, PI3-K/Akt, ERK-MAPK and JAK/STAT pathways [3, 4].

The incidence of lymphoma in the Chinese population is approximately six cases per 100 000 people [42]. We found that LMP1 expression was significantly associated with poorer survival in NHL patients, possibly due to similar mechanisms discussed above for NPC. Moreover, LMP1 has the ability to immortalize resting B lymphocytes and transform them into permanent, latently infected lymphoblastoid cell lines, which plays an important role in the etiology and the progression of the disease [3, 5, 21, 25]. We observed heterogeneity in our meta-analysis of NHL patients due to the study by Kanemitsu et al., which unlike other studies, showed improved OS was associated with LMP1 expression [13]. The results may have differed in this study because of the relatively small sample size, regional distribution, or different LMP1 variants used by Kanemitsu et al. [13].

Unlike NPC and NHL, we found no significant association between LMP1 expression and survival in HD patients. As a unique type of lymphoma, EBV-associated HD has an unbalanced distribution: a relatively higher proportion of EBV-positive cases are found among children and elderly patients, whereas young adults are usually EBV-negative. Additionally, the proportion of EBV-positive cases in Europe and North America varies from 27% to 51%, but the highest frequencies (70% to 100%) of EBV-positive cases are found in developing countries (e.g., India, South Africa) [14, 43, 44]. Therefore, the prognostic effect of LMP1 expression on HD may be affected by a variety of factors, including the patients' age and geographical location. This may explain why we found no significant association between LMP1 and survival in the entire HD patient population in our study compared with NPC or NHL patients. In fact, our subgroup analyses showed that age and geographical region do affect the prognosis of HD patients with LMP1 expression. Particularly, LMP1 expression was significantly associated with poorer OS in studies where the patients' median/mean age was ≥40 years (HR = 1.82) but had a tendency to improve OS in studies where patients' median/mean age was <40 years (HR = 0.69). In studies from Europe and North America, LMP1 expression had a tendency to worsen OS (HR = 1.42), while it improved OS in patients from other geographical regions (HR = 0.24). There are a few reasons to explain why age and geographical regions have an effect on the prognosis of HD patients with LMP1 expression. In HD, LMP1 expression may have reciprocal effects on the expressing cells. As LMP1 has antigenicity, one study found that cytotoxic T-cells infiltrated into the lesion in LMP1-positive HD cases and this was associated with tumor regression [45]. Therefore, although LMP1 may induce a malignant phenotype, it may also elicit a more effective immune response, resulting in the favorable outcomes in some HD cases [13, 35]. It is possible that there is a beneficial LMP1-induced immune response within younger people, while in older patients, with relatively lower immunocompetence, this response may be less effective [31, 39]. Similarly, in developing countries, children have a relatively high risk of being exposed to a wide spectrum of infectious agents from an early age, so HD patients from these countries may have a more efficient immune response induced by LMP1 expression [30, 37]. These factors may partly explain the different prognostic effects of LMP1 in the different age and geography groups in our study, although further studies are needed to support these findings.

Finally, while EBV is found in ∼10% of typical gastric adenocarcinomas, which accounts for up to 75 000 new cases per year [3]. There has been considerable controversy with respect to the prognostic effect of LMP1 on survival of GC patients [46]; Unfortunately, there are simply too few studies on GC patients to draw any meaningful conclusions. As only one study with a total of 63 LMP1-postive GC patients was included in our meta-analysis, the precise role of LMP1 in the prognosis of GC requires further investigation.

Our study demonstrates that LMP1 can help identify patients with EBV-associated cancers who are at a high risk for a poor clinical outcome. In addition to serving as a prognostic biomarker, our results suggest development of anti-LMP1 drugs could be a novel therapeutic strategy for NPC, NHL, and certain HD patients (i.e., depending on the patient's age and geographical location). New immunotherapy approaches, for example, administering anti-LMP1 cytotoxic T cells to target the malignant cell population that express LMP1, have been gaining interest [36, 47, 48]. In addition, inhibition of LMP1 expression by siRNA may be a good prospect; LPM1 siRNA has been shown to induce cell cycle arrest and enhance sensitivity to cisplatin in an EBV-positive NPC derived cell line [49].

The limitations of this meta-analysis should be acknowledged. First, this is a literature-based analysis and may have resulted in publication bias as predominantly positive results were reported. However, no evidence of publication bias was detected from the funnel plots. Second, we extracted all information from published data, rather than investigating individual patient data (IPD). This limited our ability to gain sufficient information for further analyses, and restrain us to examine the sources of heterogeneity using subgroup analyses in HD patients. Third, as we only focus on the prognostic effects of LMP1 in this study, and most of the eligible studies did not provide data regarding progression-free survival or disease-free survival. Therefore, we only used OS data for this meta-analysis. Although OS is currently the gold standard primary endpoint for survival analysis, further studies with IPD are still needed to evaluate the relationship between LMP1 and staging, and with other survival endpoints, including progression-free survival, disease-free survival, among others.

In conclusion, our meta-analysis shows that LMP1 expression can be used as a prognostic biomarker in NPC, NHL, and certain HD patients. This knowledge may assist in improving poor patient prognosis, and in designing novel therapeutic targets for EBV-associated cancers. Still, it should be noted that the development of cancer is multifactorial; various factors (e.g., smoking, diet, and environmental factors) may have an impact on the prognostic effect of LMP1 expression. Therefore, future studies with a large prospective design are required to evaluate multiple factors simultaneously, and confirm the clinical significance of LMP1 expression in EBV-associated cancers.

## MATERIALS AND METHODS

### Literature search strategy and study selection

This meta-analysis was conducted in accordance with the Preferred Reporting Items for Systematic Reviews and Meta-Analyses (PRISMA) statement [50]. Relevant studies published before May 2015 were identified through searching the following electronic databases: PubMed, Embase, Cochrane Library, Chinese National Knowledge Infrastructure, and Wanfang Database. The following search terms were used: 1) LMP1, latent membrane protein 1; 2) EBV, Epstein-Barr virus; 3) cancer, tumor, neoplasm, carcinoma; 4) survival, prognosis, prognostic factor. The analysis was supplemented by a manual search of reference lists of relevant review articles, and relevant books, and by correspondence with study investigators. The research work was examined without language limits.

All studies identified initially were screened by titles and/or abstracts before full text of the studies that satisfied our inclusion criteria were retrieved. The selection criteria for eligible studies in this study were: 1) the exposure of interest were cancer and LMP1; 2) the outcome of interests were overall survival (OS) associated with LMP1 status; 3) hazard ratio (HR) and the corresponding 95% confidence interval (CI) was either reported directly or there was sufficient data provided in the studies to calculate these values. If the studies based on the same patient populations were reported in different publications, only the most completed or the latest publication was included in this meta-analysis. Two researchers (YPC and WNZ) assessed the study eligibility independently, and any discrepancies were resolved by consensus.

### Data collection and extraction

Two independent investigators (YPC and WNZ) reviewed the publications and extracted the data. The following details were extracted: lead author, year of publication, country of origin, inclusion period, cancer type, study design, detection method, cutoff value detection, total number of patients, LMP1 status, median/mean age, median/mean follow-up duration, and assessments of outcomes (HR and the corresponding 95% CI of OS). If HR was not displayed directly, it was estimated according to the methods described by Parmar et al. [51].

### Quality assessment

Two independent reviewers (YPC and WNZ) used the Newcastle-Ottawa Scale (NOS) to assess the quality of all included studies [52]. This scale is an eight-item instrument to evaluate a study in three domains: selection of participants, study comparability, and the ascertainment of outcomes of interest. It uses the awarding of points, or “stars”, to compare study quality in a quantitative manner, with a maximum of nine stars. Studies with 7–8, 5–6, 4 and 0–3 stars were identified as very good, good, satisfactory or unsatisfactory in quality, respectively [53]. In this meta-analysis, all included studies were identified as very good or good in quality. To further minimize potential bias, we judged studies that received a score of ≥7 stars to be of high quality, and those that scored <7 stars to be of low quality, and performed the subgroup analysis accordingly.

### Statistical analysis

HRs for OS with 95% CIs according to the expression status of LMP1 were pooled. The OS was defined as the time from diagnosis to death from any cause or to the date of the last follow-up. HR was the only summary statistic calculated for this analysis, as it allows for censoring and considers the time to an event taking into account the whole survival time. A combined HR > 1 reflected a shorter survival for LMP1 positive patients. The significance of the pooled HR was determined by the *Z* test, and a *P*-value < 0.05 was considered statistically significant.

Heterogeneity across studies was evaluated by a χ^2^-based test; the I^2^ statistic, a quantitative measure of inconsistency across studies [54], was also calculated. Statistically significant heterogeneity was defined as a χ^2^ *P*-value < 0.10 or an I^2^ statistic >50%. The combinations of the estimated risks were computed by fixed-effect models and random-effect models. If the results were without heterogeneity, the fixed-effects model was used; if the results showed heterogeneity, the summary estimation was based on the Dersimonian and Laird random-effects model [55]. Because characteristics of populations and other confounding factors might not be consistent between studies, we conducted further subgroup analysis to explore the sources of heterogeneity.

Potential publication bias was assessed by visual inspection of the Begg and Egger funnel plots; the Begg and Egger tests were also performed at the *P*-value < 0.10 level of significance [56, 57]. All analyses were conducted using STATA version 12.0 (Stata Corporation, College Station, TX). All statistical tests were two-sided.
